# The Effect of Probiotic Treatment on Patients Infected with the H7N9 Influenza Virus

**DOI:** 10.1371/journal.pone.0151976

**Published:** 2016-03-17

**Authors:** Xinjun Hu, Hua Zhang, Haifeng Lu, Guirong Qian, Longxian Lv, Chunxia Zhang, Jing Guo, Haiyin Jiang, Beiwen Zheng, Fengling Yang, Silan Gu, Yuanting Chen, Qiongling Bao, Liang Yu, Xiawei Jiang, Qian Hu, Haiyan Shi, Hainv Gao, Lanjuan Li

**Affiliations:** 1 State Key Laboratory for Diagnosis and Treatment of Infectious Diseases, Collaborative Innovation Center for Diagnosis and Treatment of Infectious Diseases, The First Affiliated Hospital, School of Medicine, Zhejiang University, Hangzhou 310003, P.R. China; 2 Department of Infectious diseases, The First Affiliated Hospital, and college of Clinical Medicine of Henan University of Science and Technology, Luoyang, 471003, PR China; 3 Department of Geriatrics, the First Affiliated Hospital, College of Medicine, Zhejiang University, Hangzhou 310003, P.R. China; University of Illinois at Urbana-Champaign, UNITED STATES

## Abstract

**Background:**

A novel avian-origin influenza A (H7N9) virus emerged and spread among humans in Eastern China in 2013. Prophylactic treatment with antibiotics and probiotics for secondary infection is as important as antiviral treatment. This study aims to assess the ability of probiotic treatment to restore internal homeostasis under antibiotic pressure and to reduce/ameliorate the risk of secondary infections resulting from infection with the H7N9 virus.

**Methods:**

This is a retrospective study in archival samples. Between April 1 and May 10, 2013, 113 stool, sputum, and blood specimens were collected and analyzed by denaturing gradient gel electrophoresis (DGGE) to determine the composition of the patient microbiomes. Microbial diversity was calculated using Gel-Pro analyzer and Past software. Cluster analysis of DGGE pattern profiles was employed to create a phylogenetic tree for each patient, and multidimensional scaling (MDS) and principal component analysis (PCA) were performed to visualize relationships between individual lanes.

**Results:**

Five patients had secondary infections, including *Klebsiella pneumonia*, *Acinetobacter baumanii* and *Candida albicans* infection. The DGGE profiles of fecal samples obtained at different time points from the same individual were clearly different, particularly for patients with secondary infections. Shannon’s diversity index and evenness index were lower in all infected groups compared to the control group. After *B*. *subtilis* and *E*. *faecium* or *C*. *butyricum* administration, the fecal bacterial profiles of patients who had not been treated with antibiotics displayed a trend of increasing diversity and evenness. *C*. *butyricum* failed to reduce/ameliorate secondary infection in H7N9-infected patients, but administration of *B*. *subtilis* and *E*. *faecium* appeared to reduce/ameliorate secondary infection in one patient.

**Conclusion:**

H7N9 infection might decrease intestinal microbial diversity and species richness in humans. *C*. *butyricum* failed to reduce/ameliorate secondary infection in H7N9-infected patients. *B*. *subtilis* and *E*. *faecium* may also play a role in reducing/ameliorating secondary infection in these patients.

## Introduction

The China Health and Planning Commission notified the World Health Organization (WHO) of three novel human influenza infections on March 31, 2013. The infections, which were identified in individuals living in Shanghai and Anhui with onset of illness between Feb 19 and March 15, were characterized as avian influenza A H7N9 [1]. By the end of 2013, 144 people were infected, and an additional 100 individuals were infected by the end of January 2014. The novel H7N9 virus caused severe illness, including pneumonia and acute respiratory distress syndrome (ARDS), that often required admission to the ICU [2]. Extensive therapeutic interventions including antiviral treatment, oxygen therapy, mechanical ventilation, antibiotics, glucocorticoids, intravenous immunoglobulins, extracorporeal membrane oxygenation, continuous renal replacement therapy, and artificial liver support system therapy were employed. Despite these interventions, a high mortality rate among H7N9-infected patients was observed [2, 3]. Several differences between H7N9- and H5N1-infected patients have been reported, including significantly different patterns of leukopenia and thrombocytopenia and elevated levels of alanine aminotransferase, creatinine kinase, C-reactive protein, and lactate dehydrogenase. In addition, H7N9 patients were generally admitted for longer hospital stays than patients infected with either H5N1 or pandemic H1N1 (pH1N1) [4]. This increased duration of hospitalization frequently resulted in an increased incidence of secondary infections. Finally, while mammalian influenza viruses are restricted almost exclusively to respiratory tissue, in a recent fatal case of H7N9, the influenza transcript was isolated from stool samples by RT-PCR [5].

Inside the human body, an intricate homeostatic balance is maintained by a specific bacterial composition, known as the human intestinal microbiota [6]. The human microbiota is influenced by many environmental factors, including urbanization, rapid global mobility, highly processed diets, improved sanitation and hygiene, non-familial child care, and various medical interventions [7]. One specific medical intervention, antibiotics, is one of the most common and important mediators of human microbiota disturbance. The human microbiota is adversely altered by the overuse and overprescription of antibiotics, which do not distinguish between pathogenic and symbiotic microorganisms [8]. After extended antibiotic treatment, individuals may require colonization from outside the host to reassemble the microbiota [9].

Probiotic administration is a safe, inexpensive, and noninvasive strategy to mitigate the adverse effects of antibiotic misuse on the microbiota [10]. Administration of single-strain probiotics increases bacterial diversity in the gut [11]. Evidence suggests that nonpathogenic intestinal microbes not only modulate the local mucosal immune response but also have immunomodulatory effects on sites outside the gut, including the respiratory tract [12]. Through this mechanism, probiotic administration might reduce respiratory infections in healthy and hospitalized children [13] and protect against bacterial and viral infections in the GI and respiratory systems [12, 14]. Taken together, these findings indicate that prophylaxis with antibiotics and probiotics for secondary infection is as important as antiviral treatment for combating viral infection.

In this study, we used PCR-based denaturing gradient gel electrophoresis (PCR-DGGE) to monitor the intestinal bacterial composition of 15 patients infected with the emerging avian influenza A H7N9 virus. We retrospectively assessed the effects of *Clostridium butyricum*, *Bacillus subtilis* and *Enterococcus faecium* as microecological regulating agents. Specifically, we assessed the ability of these bacteria to restore internal homeostasis under antibiotic pressure and to reduce the risk of secondary infection.

## Materials and Methods

### Patients and stool specimen sampling

Volunteer patients at The First Affiliated Hospital, College of Medicine of Zhejiang University, China, were enrolled between April 1, 2013 and May 10, 2013. The Ethics Committee of the First Affiliated Hospital, College of Medicine of Zhejiang University, provided approval for this study. The reference number is 2013156. All patients provided written informed consent to participate in this study. In addition, there was a eight-year-old girl in this study, both the child and her father gave their written informed consent also.

The administration of antibiotics/probiotics was decided by the in-charge physician. There was no author involved in making decisions about patient care. And authors did not impact the treatment decisions and/or allocate patients to treament groups. So We believe that our study is a retrospective study. All samples(fecal, sputum, blood) were not collected for this study, we obtained fecal samples from the hospital’s biobank. The data on sputum and blood samples was obtained from patient medical charts. Only the data on fecal samples(DGGE) are results of scientific studies conduted by the authors.

The intestinal microecology of each patient was studied by collecting daily stool samples. The minimum number of stool samples for study inclusion was four. In addition, the study was terminated if drastic changes in an individual patient’s diet occurred. Samples were collected in sterile bags, refrigerated, immediately taken to the laboratory, aliquoted into 200 mg samples, frozen immediately in liquid nitrogen and stored at -80°C.

Fifteen patients met the inclusion criteria and completed the study, and a total of 113 specimens were obtained for DGGE analysis. Patients were classified into four groups based on therapy during follow-up: no antibiotic or probiotic treatment (Group A, 2 patients), antibiotic treatment with no probiotic treatment (Group B, 1 patient), treatment with one probiotic but no antibiotics (Group C, 5 patients), and treatment with antibiotics and one probiotic (Group D, 7 patients). The patient characteristics were collected from medical records (S1 Table).

Twenty healthy age-matched and sex-matched patients were enrolled as controls, and each provided a single stool sample. The exclusion criteria for the control group included hypertension, diabetes, obesity, metabolic syndrome, IBD, nonalcoholic fatty liver disease, coeliac disease and cancer. None withdrew or were omitted from the study, and none received antibiotics, probiotics, or prebiotics during the sampling period.

### Antibiotic and probiotic administration

CBM588 (MIYA-BM tablets, Miyarisan Pharmaceutical, Tokyo, Japan) is a probiotic agent (*Clostridium butyricum*) containing approximately 10^7^ cfu/tablet [15]. *C*. *butyricum* proliferates in the animal intestinal tract [16] and enhances the counts of lactobacilli and bifidobacteria in the human intestinal tract after the start of *H*. *pylori* eradication therapy [15]. Patient health status was considered while administering antibiotic and probiotic agents. The antibiotics used in group D subjects were piperacillin–tazobactam, tigecycline, imipenem, moxifloxacin, cefoperazone and fluconazole. Probiotics including *Bacillus subtilis* and *Enterococcus faecium* enteric-coated capsules or *C*. *butyricum* were used. Two tablets of probiotic compound were administered three times per day (~10^7^ cfu/tablet for CBM588 and 10^8^ cfu for *B*. *subtilis* and *E*. *faecium* enteric-coated capsules).

### DNA extraction

Following fecal sampling, DNA was extracted using a DNA extraction stool kit (Qiagen, Hilden, Germany) with a modified protocol for cell lysis [17]. DNA integrity was verified by agarose gel electrophoresis and UV-light photography with ethidium bromide staining.

### PCR amplification of the 16S rDNA V3 region

The 16S rDNA V3 region was amplified using a hot-start touchdown protocol with primers specific for conserved regions of the 16S rRNA gene [18]. The reaction mixture contained 2 μL of genomic DNA, 25 pmol of each primer, 4 μL of dNTPs, 5 μL of 10х Ex Taq buffer, 0.5 μL of TaKaRa Ex Taq polymerase (TaKaRa, Dalian, China), and sterile deionized water to a total volume of 50 μL. To minimize heteroduplex formation, five-cycle reconditioning PCR was conducted with 5 μL of amplification mixture in a fresh reaction mixture as previously described [19]. Profiles were generated using a TProfessional Thermocycler (Biometra, Göttingen, Germany). Amplified products were confirmed by agarose gel electrophoresis (1%), and their concentrations were measured using a NanoDrop ND-1000 spectrophotometer (Nano-Drop Technologies, Wilmington, DE, USA). All amplified products were stored at -20°C until DGGE analysis.

### Denaturing gradient gel electrophoresis profiling

Parallel DGGE analysis was performed using a D-Code universal mutation detection system apparatus (16 x 18 x 1.5 mm; Bio-Rad, Hercules, CA). Sequence-specific separation of the PCR fragments was performed in 8% polyacrylamide (acrylamide-N,N’ bisacrylamide; 37.5:1 [wt/vol]) gels in 1x TAE buffer (40 mM Tris, 20 mM glacial acetic acid, 1 mM EDTA, pH 8.0). Denaturing gels were prepared with a 35% to 75% gradient of urea and formamide increasing in the direction of electrophoresis. The 100% denaturing solution contained 40% (vol/vol) formamide and 7 M urea. Twenty microliters of PCR product was added per lane, and samples were resolved by electrophoresis at a constant voltage of 68 V at 60°C for approximately 16 h. Following electrophoresis, the gels were stained with SYBR Green I (Sigma-Aldrich, Castle Hill, Australia) and photographed. Each DGGE gel was normalized using a randomly selected standard reference in either the middle or at one end of the gel.

### Comparative analysis of the DGGE profiles

The DGGE profiles were digitally processed with BioNumerics software version 6.01 (Applied Maths, St-Martens-Latem, Belgium) in a multistep procedure following the manufacturer`s instructions. Profiles were compared using the band-matching tool, and uncertain bands were included in the position tolerance settings. The parameters for allocating band classes were as previously described by Joossens *et al* [20]. The UPGMA (unweighted pair-group method with arithmetic means) method based on the Dice similarity coefficient (band based) was used to perform cluster analysis of the DGGE pattern [21]. Multidimensional scaling (MDS) and principal component analysis (PCA) were performed as previously described (BioNumerics). MDS is an optimized 3D representation of the similarity matrix, and these similarities were calculated as a best estimate using the Euclidean distance between two gel lanes to provide a convenient visual interpretation. PCA is another method to visualize relationships among lanes by reorienting the plot to maximize the variation among lanes along the first two principal components.

### Sequencing of DGGE bands

Predominant bands were excised from the denaturing gradient gels, placed in sterile Eppendorf tubes, washed three times with TE buffer (10 mM Tris-HCl, 1 mM EDTA, pH 8.0), and dissolved by incubation in 50 μL of TE buffer for 30 min at 80°C. Five microliters of buffer solution was used as a template for PCR re-amplification with the universal bacterial primers F357+GC clamp and R518. Amplicons were analyzed by DGGE and excised until a single band was obtained. Amplicons without GC clamps were purified using the QIAquick PCR purification kit (Qiagen, Hilden, Germany), ligated with pGEM-T Easy Vector (Promega, Madison, WI, USA), and transformed into competent *E*. *coli* DH5 cells. Positive clones were verified and sequenced (Invitrogen, Shanghai, China). Homology was identified by conducting a BLAST search of the GenBank DNA database [20]. Reference sequences of phylogenetic neighbor species (up to 98% similarity) were included for clustering analysis using multiple sequence alignment with K-Lite Mega Codec Pack 5.05 [22].

### Statistical analysis

Statistical analyses were performed with the software package SPSS for Windows (version 18.0; SPSS, Inc., Chicago, IL,USA). Statistical significance among groups was analyzed by one-way ANOVA analysis. Statistical significance was set at p< 0.05.

## Results

### Retrospective analysis of antibiotic and probiotic use and infection

We first assessed each study group for secondary infection following H7N9 infection. Analysis of sputum samples revealed that five patients (C3, D1, D2, D4 and D6) had secondary infections, including *Klebsiella pneumonia*, *Acinetobacter baumanii* and *Candida albicans* infection. The secondary infections were resolved in three patients (C3, D4 and D6; S1 Table). We also monitored in real time the concentration of C-reactive protein (CRP), an early indicator of infectious or inflammatory conditions, and observed a decrease in CRP levels in twelve patients (A1, A2, B1, C1, C2, C3, C4, C5, D4, D5, D6 and D7; S1 Table).

### Fecal microbiome profiles

**Fig 1:**
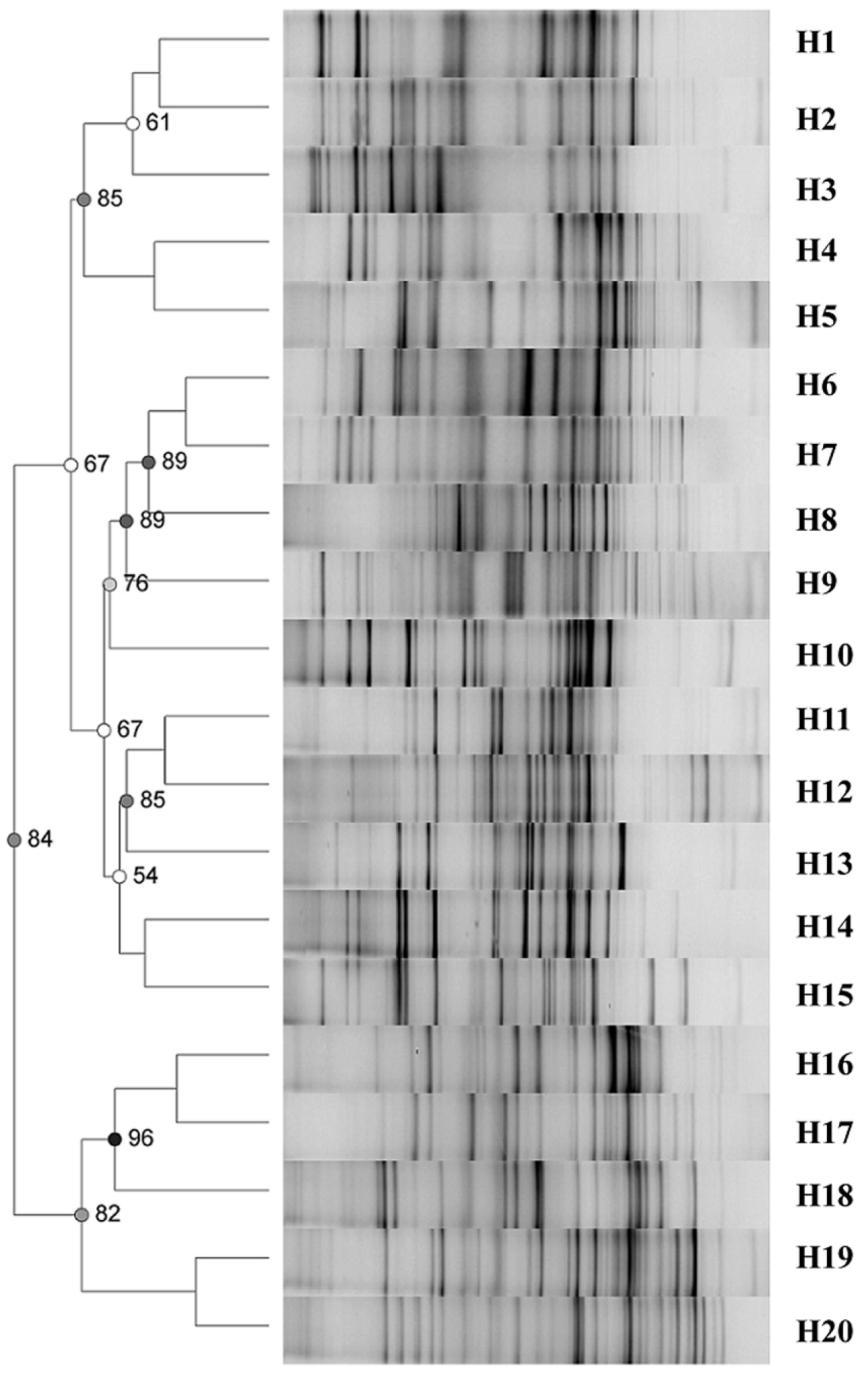
Clustering of DGGE profiles obtained with V3 universal primers for 20 controls. Cluster analysis was performed using Dice’s coefficient and UPGMA. Metric scale denotes the degree of similarity. The DGGE profiles of the intestinal flora of the healthy control group revealed high diversity among the individuals.

**Fig 2:**
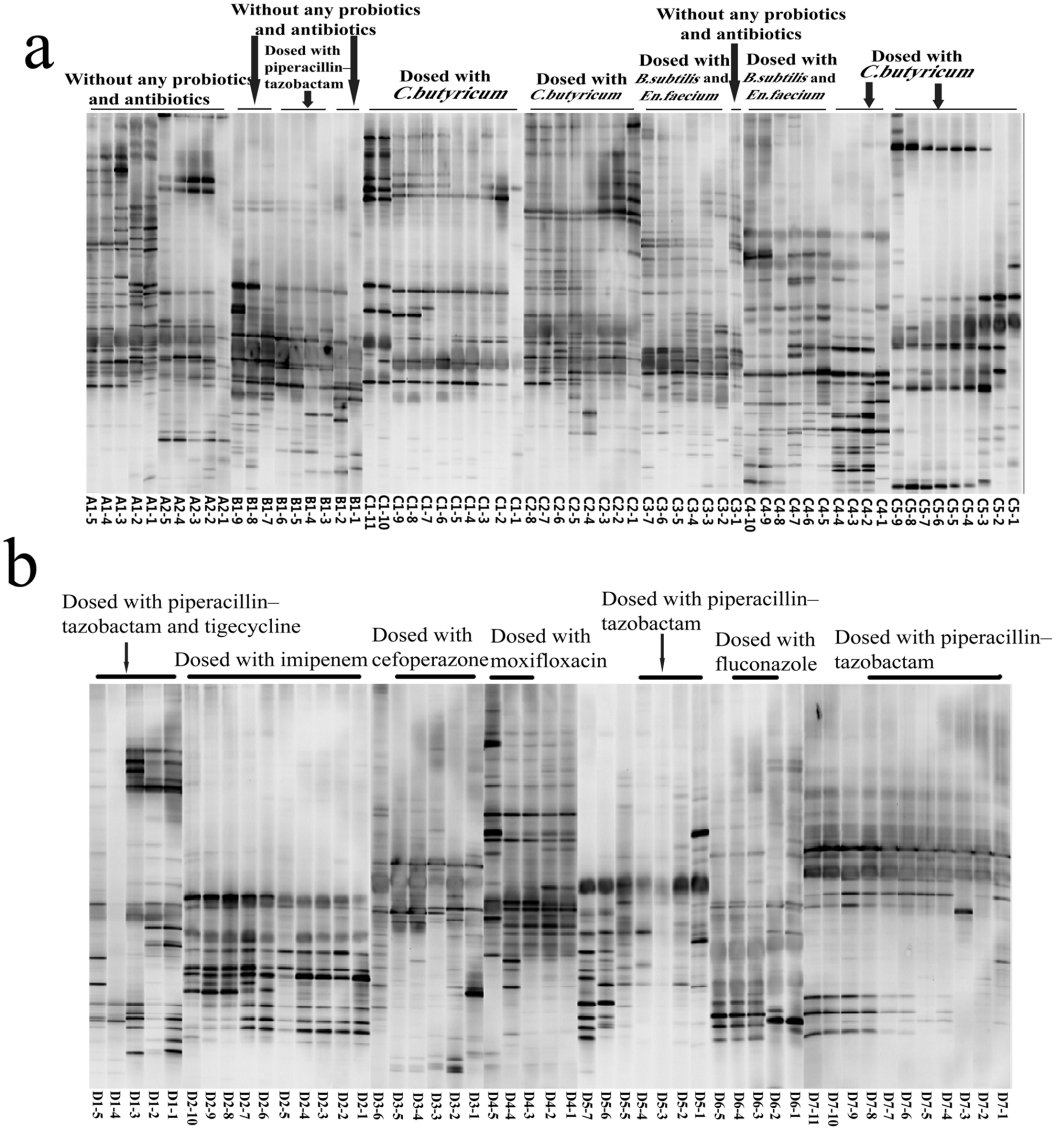
DGGE profiles of the predominant intestinal bacteria in H7N9-infected patients. **(a)** DGGE profiles of fecal bacteria in A, B and C groups. **(b)** Differences in the DGGE profiles of fecal samples taken at different time points from the same individual were apparent, particularly for patients D1, D2, D4, D5 and D7. This result suggests temporal instability in the predominant bacterial population in H7N9-infected patients with secondary infection.

**Fig 3:**
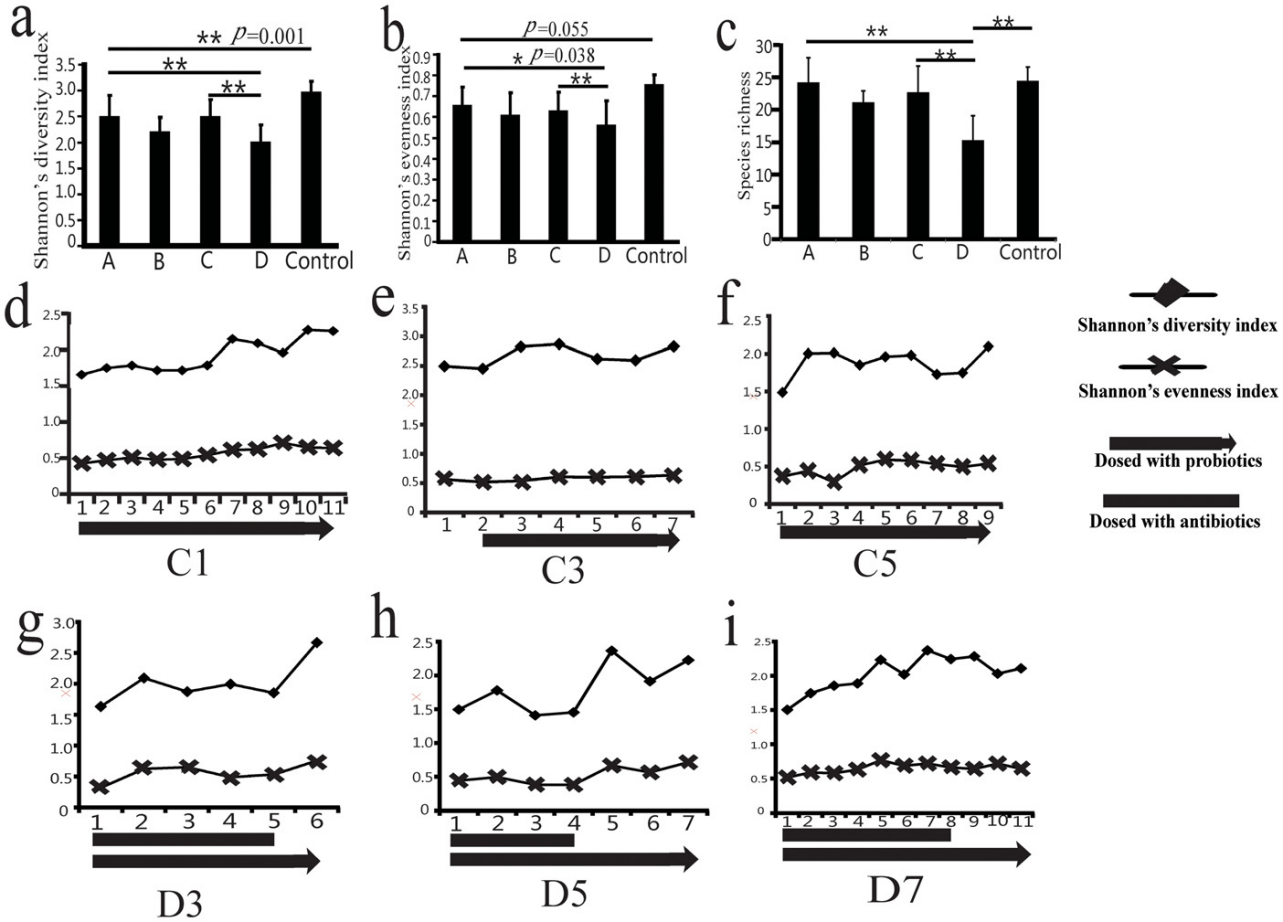
Intestinal microbial diversity comparison. **(a)** Shannon’s diversity index comparison. **(b)** Shannon’s evenness index comparison. **(c)** Species richness comparison. Shannon’s diversity index and Shannon’s evenness index were lower in all infected groups compared to the control group. A decreased Shannon’s diversity, evenness and species richness in group D patients compared to patients in the A and C groups. * P<0.05, ** P<0.01 **(d)** The changes in Shannon’s diversity and evenness in patient C1. X axis, time of sampling. **(e)** The changes in Shannon’s diversity and evenness in patient C3. **(f)** The changes in Shannon’s diversity and evenness in patient C5. **(g)** The changes in Shannon’s diversity and evenness in patient D3. After *B*. *subtilis* and *E*. *faecium* or *C*. *butyricum* administration, the fecal bacterial profiles of patients without antibiotics displayed a trend of increasing diversity and evenness. **(h)** The changes in Shannon’s diversity and evenness in patient D5. **(i)** The changes in Shannon’s diversity and evenness in patient D7. The results showed that a trend toward increasing diversity and evenness in D3, D5 and D7 after antibiotic cessation.

The DGGE profiles of the intestinal flora of the healthy control group were highly diverse (Fig 1). The DGGE profiles of H7N9-infected patients revealed shifts in the composition of intestinal microbial communities (Fig 2a and 2b). To quantify these differences, we first calculated the intensity of each band in each lane of the DGGE profiles using Gel-Pro analyzer software. We then analyzed the microbial diversity using Past software. The results demonstrated that Shannon’s diversity index and Shannon’s evenness index were lower in all infected groups compared to the control group (Fig 3a–3c). These results suggest that H7N9 infection might decrease intestinal microbial diversity and species richness in humans.

As shown in Fig 3a, 3b and 3c, a decreased Shannon’s diversity, evenness and species richness in group D patients compared to patients in the A and C groups. Moreover, differences in the PCR-DGGE profiles of fecal samples taken from group D subjects at different time points were observed (Fig 2b). These data suggest temporal instability in the predominant bacterial populations of H7N9-infected patients with secondary infection.

After *B*. *subtilis* and *E*. *faecium* or *C*. *butyricum* administration, there was no significance difference between group A and C (Fig 3a–3c). However, the fecal bacterial profiles of patients who had not been treated with antibiotics displayed a trend of increasing diversity and evenness(C1, C3 and C5; Fig 3d–3f). Moreover, the use of probiotics did not show significant difference to increase the diversity in patients had secondary bacterial infections(D1, D2, D4 and D6). But the results showed that a trend toward increasing diversity and evenness in some group D patients after antibiotic cessation(D3, D5 and D7; Fig 3g–3i).

**Fig 4:**
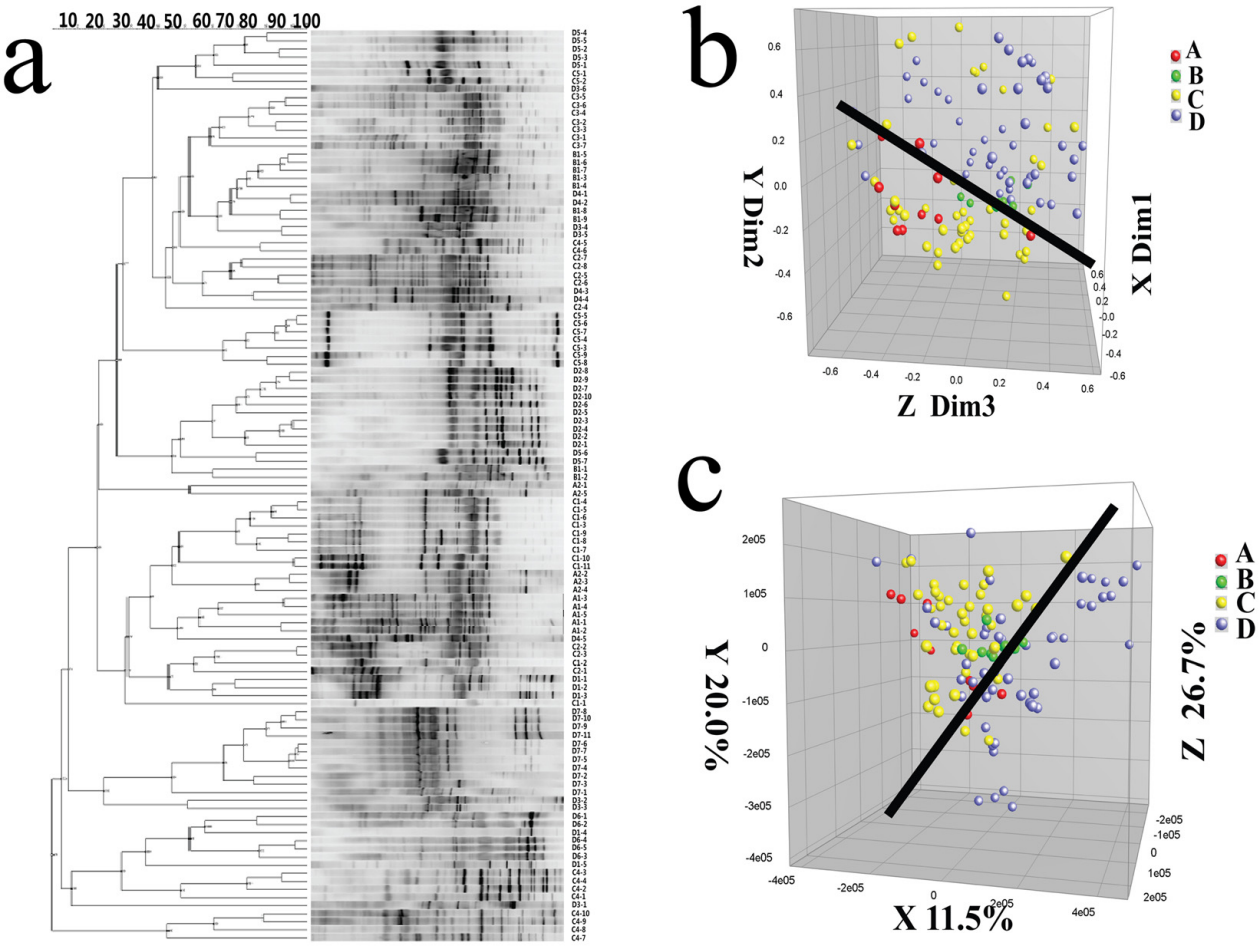
Cluster analysis of the DGGE profiles of the predominant fecal bacteria of 15 patients in follow-up samples. Clustering was performed using Dice’s coefficient and UPGMA. **(a)** Cluster analysis of the DGGE profiles from the different groups. The metric scale denotes the degree of similarity. **(b)** MDS analysis of the cluster shown in (a). The plot is an optimized 3D representation of the similarity matrix obtained from BioNumerics software, and the x-, y-, and z-axes separately represent three different dimensions: Dim 1, Dim 2, and Dim 3. The Euclidean distance between two points reflects similarity. **(c)** PCA of fecal microbiota based on the DGGE fingerprinting shown in (a). The plot is reoriented to maximize variation among lanes along the first three principal components (the contributions 11.5, 20.0 and 26.7, respectively) obtained from BioNumerics software.

**Fig 5:**
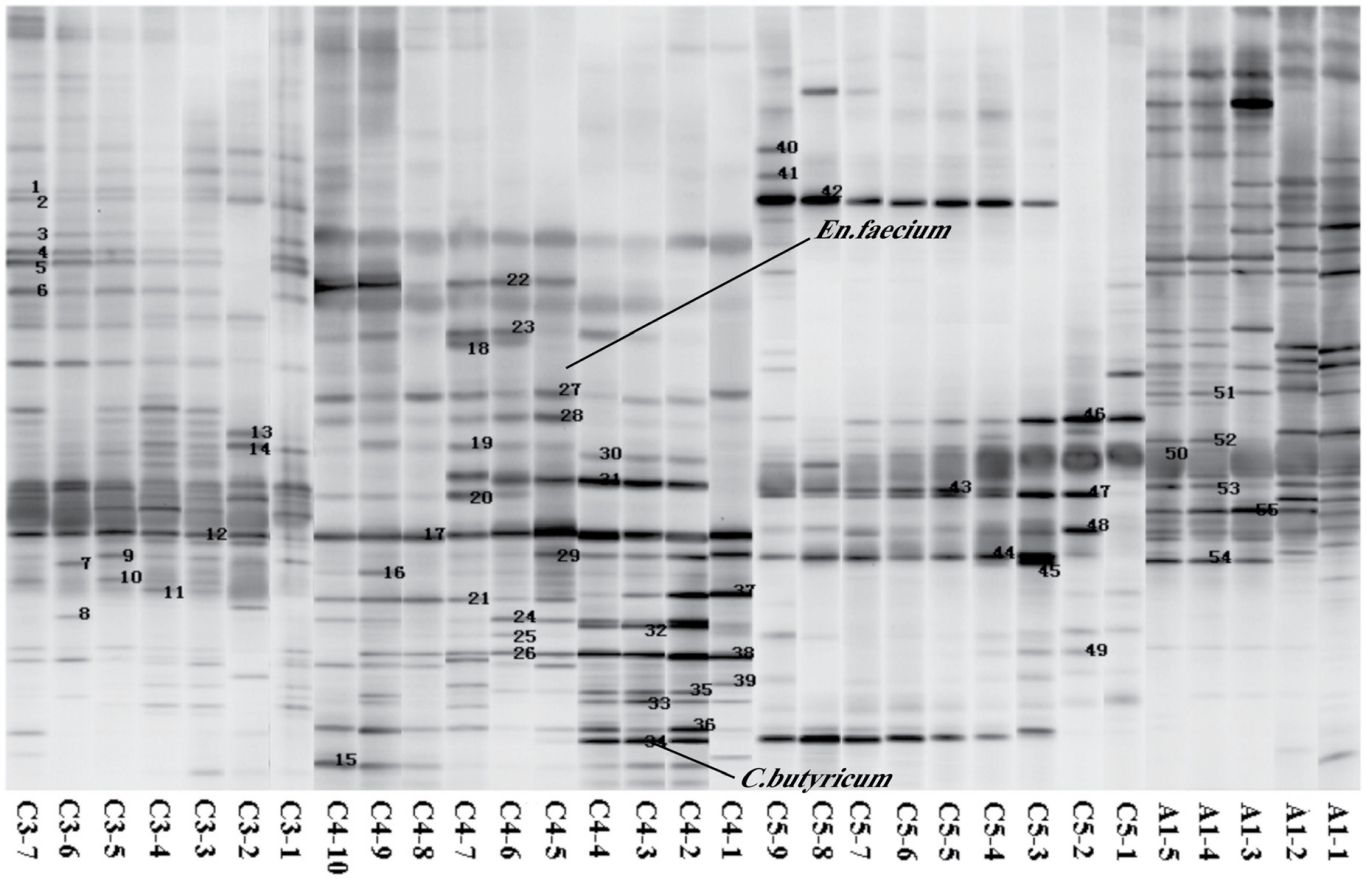
The numbers of predominant bands were excised from the DGGE gels. Each band represents a bacterial clone. Band numbers (corresponding to Fig 6 band classes) indicated the position of bands excised for sequence analyses (e.g. ‘“20”‘ means band 20).

**Fig 6:**
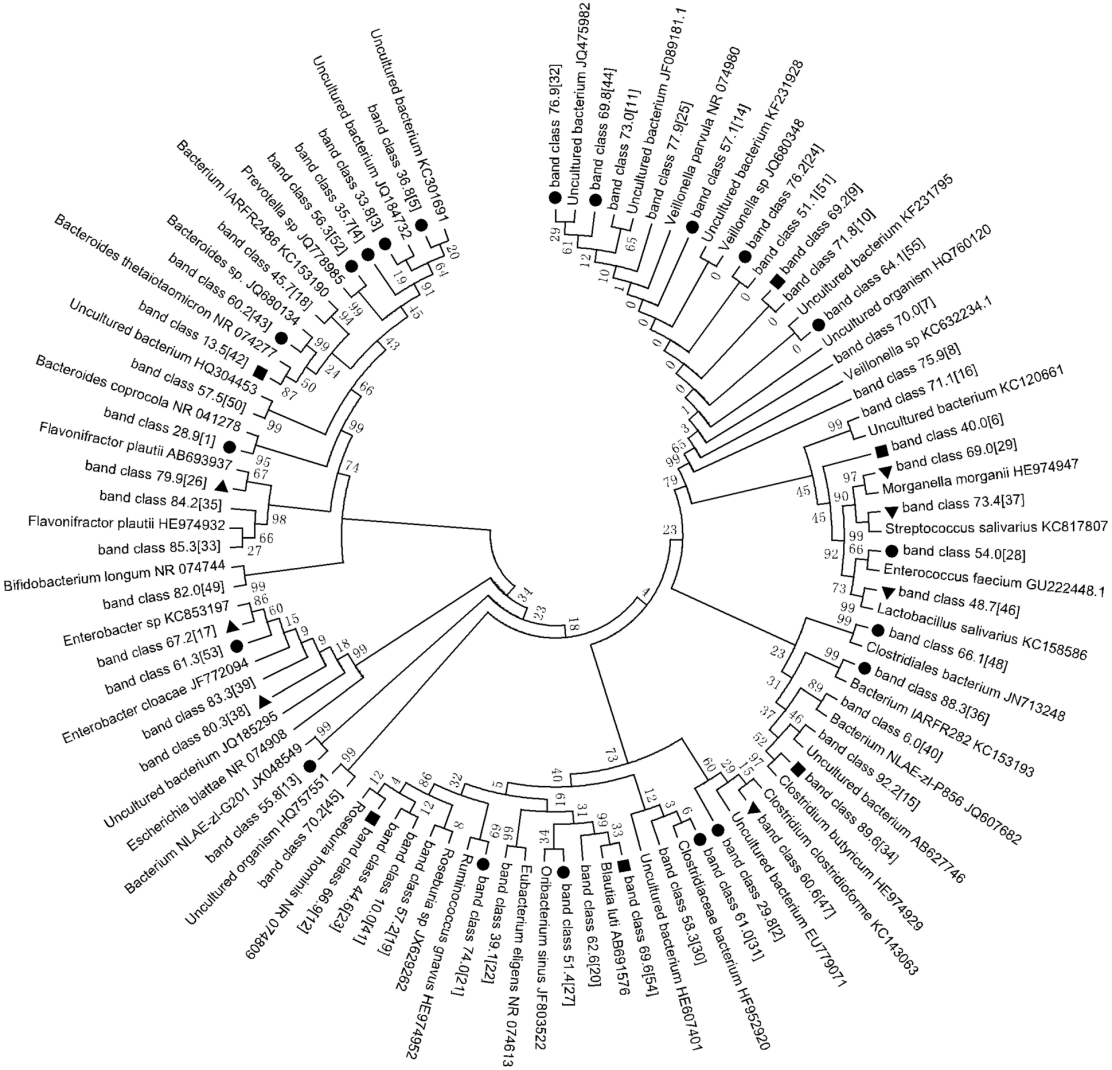
Phylogenetic tree analysis of DGGE profiles. Phylogenetic tree of sequences constructed using the neighbor-joining method based on the DGGE profiles. The fragment sequences were named for their positions in the gels using the band-matching tool with BioNumerics software version 6.01 (Applied Math). Twenty-one band classes, indicated by black spots, displayed little variation in intensity in the follow-up samples. Seven band classes, indicated by black triangles, exhibited a increase in intensity in the follow-up samples. Six band classes, indicated by black squares, exhibited an decrease in the follow-up samples. The plot was generated using MEGA5.1 software.

We then identified differences in the microbial structures of patients in the A, B, and C groups compared to the D group by MDS (Dim 1, Dim 2, and Dim 3) and PCA axis X/Y/Z (contribution rate: 11.5%, 20.0 and 26.7%, respectively; Fig 4b and 4c). Here, the difference between two data points was directly related to the difference in microbial sample composition. These results indicate that the effects of treatment with a combination of antibiotics and probiotics differed from the effects of treatment with either option separately. We then constructed a phylogenetic tree based on the DGGE band sequences to clarify the phylogenetic relationships between intestinal bacterial species to identify key bacterial composition changes in H7N9-infected patients (Figs 5 and 6). Of 55 PCR-DGGE bands, 44 were identified. To assign bands to a bacterial species or phylotype, DNA was purified by band class from at least two different samples, sequenced, and tentatively assigned to a bacterial species or phylotype based on the highest sequence similarity match to GenBank sequences by BLAST analysis (98–100%). We then compared and analyzed changes in each band among the different groups based on the intensity of each band. Twenty-one band classes exhibited little variation in intensity, both were assigned to the phylum Firmicutes (Fig 6). Five band classes exhibited an distinctly increase after the probiotics administration. Three band classes exhibited a distinctly decrease in intensity after the probiotics administration (S2 Table).

## Discussion

The use of and interest in probiotic treatments and microbiome composition have increased in recent years. In this study, we investigated probiotic treatment as a means to restore infection-induced perturbations in the commensal gut microbiome of infected patients. The intestinal microbiota is considered an important micro-ecosystem that has a symbiotic relationship with the body, equivalent to a major metabolic "organ" [23–25]. The human intestinal tract is colonized with microorganisms from birth and contains complex and diverse bacterial communities, providing a unique microbiota that is relatively stable over time [26, 27]. The intestinal microbiota plays an important role in human physiology, including gut maturation, colonization resistance, various metabolic processes, regulation of intestinal epithelial proliferation and modulation of the mucosal and systemic immune responses [28–31]. The intricate balance between the colonic microbiota and the effects of a dysbiotic microbiota and its metabolic products on host physiology [32, 33] and disease processes [8, 34–36] have been established. Perhaps the most interesting result in our study was the H7N9 infection decreases human intestinal microbial diversity and species richness. Moreover, after *B*. *subtilis* and *E*. *faecium* or *C*. *butyricum* administration, there was no significance difference between group A and C. But the fecal bacterial profiles of patients who had not been treated with antibiotics displayed a trend of increasing species richness, diversity and evenness, even in the absence of antibiotic effects. For most patients with H7N9 infection in the present study, antibiotics were used in the early stage of the disease. Some patients received prophylaxis against healthcare-associated infections. Our findings indicate that antibiotic treatment can lead to not only a reduction in species diversity but also long-term perturbations in the commensal gut microbiome, with detrimental health effects. Most patients in our study were treated with antibiotics during the early stages of disease, and some received repeated antibiotic therapy for secondary infections. This study sheds light on the need for effective strategies, including probiotics, to decrease antibiotic overuse and its effect on influenza infection to improve host health [37]. Therefore, probiotics should be administered often during and after antibiotic treatment.

In this study, we observed a decreased Shannon’s diversity index and species richness in group D patients compared to patients in the A and C groups. These results indicate that *C*. *butyricum* failed to reverse the disturbances in the gut microbiome induced by antibiotic treatment of H7N9-infected patients. However, we observed a trend toward increasing diversity and evenness in some group D patients after antibiotic cessation, indicating the need for continued probiotic treatment following antibiotic cessation [38, 39]. The microbiota appears to be profoundly altered by frequent prior antibiotic use, and much longer probiotic treatment might be required to reverse these effects.

In this study, we did not observe either a marked increase in CRP levels or bacteremia and pneumonia in the patients treated with probiotics. These data demonstrate that the probiotics were safe and did not induce inflammatory effects.

Bacterial adherence to the surface of cells and the respiratory tract can be enhanced by influenza virus infection, leading to increased secondary respiratory tract bacterial infections [40]. In addition, probiotics have protective effects against bacterial and viral infections in the GI and respiratory systems, and administration of probiotics has been associated with a lower incidence of ventilator-associated pneumonia [14, 41], reduced respiratory infections in healthy and hospitalized children [13, 42], and reduced duration of common cold infections [43]. Therefore, the another aim of probiotic treatment was to reduce/ameliorate secondary infection in these patients. Unfortunately, our data indicate that *C*. *butyricum* failed to reduce/ameliorate secondary infection in H7N9-infected patients. But administration of *B*. *subtilis* and *E*. *faecium* appeared to reduce/ameliorate secondary infection in one patient (C3), indicate that *B*. *subtilis* and *E*. *faecium* may also play a role in reducing/ameliorating secondary infection. Future studies are requierd to support such claims and to uncover the mechanisms. While our findings are compelling, the number of patients in our study was small. A more robust, complete clinical trial is needed to provide further details. In addition, the urgency of H7N9 infection necessitated the use of diverse and extensive medication, which might have affected the composition of the microbiota.

## Supporting Information

S1 Table Patient information at the time of sampling. (DOCX)

S2 Table Name of microbes distinctly changed after the probiotics administration. (DOC)
