# Supplementary material for: The Effect of Probiotic Treatment on Patients Infected with the H7N9 Influenza Virus
Source: PLoS One. 2016 Mar 17;11(3):e0151976. doi: 10.1371/journal.pone.0151976 (PMC4795712; doi:10.1371/journal.pone.0151976)
Supplement: S1 Table — (DOCX) [file pone.0151976.s001.docx]

**Table S1** Patients information at the time of sampling

| Patient | Sex | Age | Time of sampling  (days after admission) | WBC | CRP | Antimicrobial agents | Probiotics | Sputum-isolated secondary infections |
| --- | --- | --- | --- | --- | --- | --- | --- | --- |
| A1-1 | F | 36 | 2 | 2.8 | 7.4 | None | None | None |
| A1-2 |  |  | 4 | 4.1 | 1.9 | None | None | None |
| A1-3 |  |  | 11 | 4.7 | 1 | None | None | None |
| A1-4 |  |  | 13 | 10.7 | 1.4 | None | None | None |
| A1-5 |  |  | 15 | 9.6 | 0.7 | None | None | None |
| A2-1 | F | 8 | 2 | 4.4 | 11.8 | None | None | None |
| A2-2 |  |  | 4 | 3.7 | 2.4 | None | None | None |
| A2-3 |  |  | 5 | 4.6 | -- | None | None | None |
| A2-4 |  |  | 6 | 4.6 | -- | None | None | None |
| A2-5 |  |  | 7 | 4.9 | 1 | None | None | None |
| B1-1 | M | 58 | 1 | 2.4 | 50 | None | None | None |
| B1-2 |  |  | 3 | 3.1 | 45.4 | None | None | None |
| B1-3 |  |  | 7 | 6.4 | 6.1 | piperacillin–tazobactam | None | None |
| B1-4 |  |  | 8 | -- | -- | piperacillin–tazobactam | None | None |
| B1-5 |  |  | 9 | -- | -- | piperacillin–tazobactam | None | None |
| B1-6 |  |  | 10 | 6.2 | 1.4 | piperacillin–tazobactam | None | None |
| B1-7 |  |  | 11 | -- | -- | None | None | None |
| B1-8 |  |  | 14 | 5.7 | 1 | None | None | None |
| B1-9 |  |  | 15 | 6.8 | 1 | None | None | None |
| C1-1 | M | 43 | 1 | 2.7 | 53.4 | None | *C. butyricum* | None |
| C1-2 |  |  | 5 | 3.5 | 13.8 | None | *C. butyricum* | None |
| C1-3 |  |  | 6 | 4 | 8.5 | None | *C. butyricum* | None |
| C1-4 |  |  | 7 | 4.8 | 5.8 | None | *C. butyricum* | None |
| C1-5 |  |  | 8 | 4.3 | 4.1 | None | *C. butyricum* | None |
| C1-6 |  |  | 9 | 4.4 | 2 | None | *C. butyricum* | None |
| C1-7 |  |  | 10 | 3.8 | 2.7 | None | *C. butyricum* | None |
| C1-8 |  |  | 11 | -- | -- | None | *C. butyricum* | None |
| C1-9 |  |  | 12 | -- | -- | None | *C. butyricum* | None |
| C1-10 |  |  | 13 | -- | -- | None | *C. butyricum* | None |
| C1-11 |  |  | 15 | 4.5 | 1.3 | None | *C. butyricum* | None |
| C2-1 | F | 68 | 2 | 4.3 | 15.1 | None | *C. butyricum* | None |
| C2-2 |  |  | 3 | 5.2 | 21.7 | None | *C. butyricum* | None |
| C2-3 |  |  | 4 | 6.3 | 49 | None | *C. butyricum* | None |
| C2-4 |  |  | 5 | 8.1 | 51.9 | None | *C. butyricum* | None |
| C2-5 |  |  | 6 | 6.9 | 56 | None | *C. butyricum* | None |
| C2-6 |  |  | 9 | 6.9 | -- | None | *C. butyricum* | None |
| C2-7 |  |  | 11 | -- | -- | None | *C. butyricum* | None |
| C2-8 |  |  | 13 | 6.5 | 4 | None | *C. butyricum* | None |
| C3-1 | M | 55 | 1 | 1.6 | 46.2 | None | None | *C.albicans* |
| C3-2 |  |  | 4 | 6.9 | 3 | None | *B. subtilis* , *E. faecium* | *C.albicans* |
| C3-3 |  |  | 6 | -- | 2.4 | None | *B. subtilis* , *E. faecium* | None |
| C3-4 |  |  | 7 | 4.8 | 0.8 | None | *B. subtilis* , *E. faecium* | None |
| C3-5 |  |  | 8 | -- | -- | None | *B. subtilis* , *E. faecium* | None |
| C3-6 |  |  | 9 | -- | -- | None | *B. subtilis* , *E. faecium* | None |
| C3-7 |  |  | 10 | 5.1 | 2 | None | *B. subtilis* , *E. faecium* | None |
| C4-1 | F | 58 | 1 | 3.4 | 18.3 | None | *C. butyricum* | None |
| C4-2 |  |  | 3 | 5 | -- | None | *C. butyricum* | None |
| C4-3 |  |  | 4 | 5.2 | 4.1 | None | *C. butyricum* | None |
| C4-4 |  |  | 5 | 7.5 | 3.5 | None | *C. butyricum* | None |
| C4-5 |  |  | 8 | 11.7 | 15.1 | None | *B. subtilis* , *E. faecium* | None |
| C4-6 |  |  | 9 | 13.9 | 4.5 | None | *B. subtilis* , *E. faecium* | None |
| C4-7 |  |  | 10 | -- | -- | None | *B. subtilis* , *E. faecium* | None |
| C4-8 |  |  | 11 | 8.9 | 4.1 | None | *B. subtilis* , *E. faecium* | None |
| C4-9 |  |  | 12 | -- | -- | None | *B. subtilis* , *E. faecium* | None |
| C4-10 |  |  | 13 | -- | -- | None | *B. subtilis* , *E. faecium* | None |
| C5-1 | M | 58 | 2 | 3.3 | 36.1 | None | *C. butyricum* | None |
| C5-2 |  |  | 3 | 5.6 | -- | None | *C. butyricum* | None |
| C5-3 |  |  | 4 | 7.0 | 7.2 | None | *C. butyricum* | None |
| C5-4 |  |  | 5 | 5.8. | 3 | None | *C. butyricum* | None |
| C5-5 |  |  | 6 | 5.7 | 2.4 | None | *C. butyricum* | None |
| C5-6 |  |  | 7 | 6.2 | 1.5 | None | *C. butyricum* | None |
| C5-7 |  |  | 8 | -- | -- | None | *C. butyricum* | None |
| C5-8 |  |  | 10 | -- | -- | None | *C. butyricum* | None |
| C5-9 |  |  | 12 | 4.4 | 3.9 | None | *C. butyricum* | None |
| D1-1 | M | 68 | 5 | 8.7 | 12.4 | piperacillin–tazobactam tigecycline | *C. butyricum* | *K. pneumonia* |
| D1-2 |  |  | 6 | 10.1 | 103.4 | piperacillin–tazobactam tigecycline | *C. butyricum* | *K. pneumonia* |
| D1-3 |  |  | 14 | 7.9 | 247.5 | piperacillin–tazobactam tigecycline | *C. butyricum* | *K. pneumonia* |
| D1-4 |  |  | 15 | 7.6 | 230 | piperacillin–tazobactam tigecycline | *C. butyricum* | *K. pneumonia* |
| D1-5 |  |  | 16 | 9.7 | 194.2 | piperacillin–tazobactam tigecycline | *C. butyricum* | *K. pneumonia* |
| D2-1 | M | 68 | 13 | 6.7 | 9.7 | [imipenem](app:ds:imipenem) | *C. butyricum* | *A. baumani* |
| D2-2 |  |  | 14 | 5.8 | 23.3 | [imipenem](app:ds:imipenem) | *C. butyricum* | *A. baumani* |
| D2-3 |  |  | 16 | 4.3 | 36 | [imipenem](app:ds:imipenem) | *C. butyricum* | *A. baumani* |
| D2-4 |  |  | 17 | 4.3 | 14.4 | [imipenem](app:ds:imipenem) | *C. butyricum* | *A. baumani* |
| D2-5 |  |  | 18 | 3.4 | -- | [imipenem](app:ds:imipenem) | *C. butyricum* | *A. baumani* |
| D2-6 |  |  | 21 | 4.8 | 44.5 | [imipenem](app:ds:imipenem) | *C. butyricum* | *A. baumani* |
| D2-7 |  |  | 24 | 7.7 | 57.6 | [imipenem](app:ds:imipenem) | *C. butyricum* | *A. baumani* |
| D2-8 |  |  | 26 | 5.8 | 44.9 | [imipenem](app:ds:imipenem) | *C. butyricum* | *A. baumani* |
| D2-9 |  |  | 27 | 7.0 | 10.2 | [imipenem](app:ds:imipenem) | *C. butyricum* | *A. baumani* |
| D2-10 |  |  | 28 | 9.9 | 3.2 | [imipenem](app:ds:imipenem) | *C. butyricum* | *A. baumani* |
| D3-1 | M | 39 | 3 | 12.8 | 4.2 | cefoperazone | *C. butyricum* | None |
| D3-2 |  |  | 4 | 7.9 | 1.9 | Cefoperazone | *C. butyricum* | None |
| D3-3 |  |  | 5 | 4 | 1.1 | Cefoperazone | *C. butyricum* | None |
| D3-4 |  |  | 8 | 4.9 | 0.4 | Cefoperazone | *C. butyricum* | None |
| D3-5 |  |  | 9 | 3.6 | 5.2 | Cefoperazone | *C. butyricum* | None |
| D3-6 |  |  | 11 | 5.1 | 2.6 | None | *C. butyricum* | None |
| D4-1 | M | 38 | 3 | 9 | 1.2 | None | *C. butyricum* | *K. pneumonia* |
| D4-2 |  |  | 4 | 9.5 | 0.8 | None | *C. butyricum* | *K. pneumonia* |
| D4-3 |  |  | 5 | 7.5 | 2.7 | moxifloxacin | *C. butyricum* | None |
| D4-4 |  |  | 9 | 3.2 | 3.2 | Moxifloxacin | *C. butyricum* | None |
| D4-5 |  |  | 13 | 2.8 | 0.9 | Moxifloxacin | *C. butyricum* | None |
| D5-1 | F | 55 | 1 | 2.9 | 75 | piperacillin–tazobactam | *C. butyricum* | None |
| D5-2 |  |  | 4 | 1.8 | 39.4 | piperacillin–tazobactam | *C. butyricum* | None |
| D5-3 |  |  | 5 | 20.7 | 12.6 | piperacillin–tazobactam | *C. butyricum* | None |
| D5-4 |  |  | 6 | 11.7 | 5.9 | piperacillin–tazobactam | *C. butyricum* | None |
| D5-5 |  |  | 9 | -- | 1 | None | *C. butyricum* | None |
| D5-6 |  |  | 11 | -- | -- | None | *C. butyricum* | None |
| D5-7 |  |  | 13 | 4.6 | 1.9 | None | *C. butyricum* | None |
| D6-1 | F | 80 | 3 | 7.2 | 35.7 | None | *C. butyricum* | *C.albicans* |
| D6-2 |  |  | 5 | 5.9 | 60.1 | fluconazole | *C. butyricum* | None |
| D6-3 |  |  | 10 | 9.2 | 64.5 | fluconazole | *C. butyricum* | None |
| D6-4 |  |  | 14 | 7.0 | 27.9 | fluconazole | *C. butyricum* | None |
| D6-5 |  |  | 16 | 7.5 | None | None | *C. butyricum* | None |
| D7-1 | M | 75 | 7 | 9.9 | 29.8 | piperacillin–tazobactam | *C. butyricum* | None |
| D7-2 |  |  | 8 | 9.3 | 24.3 | piperacillin–tazobactam | *C. butyricum* | None |
| D7-3 |  |  | 9 | 10.1 | -- | piperacillin–tazobactam | *C. butyricum* | None |
| D7-4 |  |  | 10 | 13 | 45.1 | piperacillin–tazobactam | *C. butyricum* | None |
| D7-5 |  |  | 11 | 12.2 | 56.7 | piperacillin–tazobactam | *C. butyricum* | None |
| D7-6 |  |  | 12 | 12.9 | 48.9 | piperacillin–tazobactam | *C. butyricum* | None |
| D7-7 |  |  | 13 | -- | 25.9 | piperacillin–tazobactam | *C. butyricum* | None |
| D7-8 |  |  | 15 | 11.1 | 14.9 | piperacillin–tazobactam | *C. butyricum* | None |
| D7-9 |  |  | 16 | -- | 24.3 | None | *C. butyricum* | None |
| D7-10 |  |  | 18 | 9.8 | 13 | None | *C. butyricum* | None |
| D7-11 |  |  | 20 | 9.2 | 11 | None | *C. butyricum* | None |
