# Supplementary material for: The Effect of Probiotic Treatment on Patients Infected with the H7N9 Influenza Virus
Source: PLoS One. 2016 Mar 17;11(3):e0151976. doi: 10.1371/journal.pone.0151976 (PMC4795712; doi:10.1371/journal.pone.0151976)
Supplement: S2 Table — (DOC) [file pone.0151976.s002.doc]

**Table 2** Name of microbes distinctly changed after the probiotics administration.

| **Band** | **Band Class (%)** | **Closest BLAST match** | **Identity (%)** | **Accession no.** | **After the probiotics administration** |
| --- | --- | --- | --- | --- | --- |
| 17 | 67.2 | *Enterobacter* sp. | 96 | KC853197.1 | **↑** |
| 37 | 73.4 | *Streptococcus* *salivarius* | 98 | KC817807.1 | **↑** |
| 38 | 80.3 | *Enterobacter cloacae* | 99 | JF772094.1 | **↑** |
| 46 | 48.7 | *Lactobacillus salivarius* | 95 | KC158586.1 | **↑** |
| 47 | 60.6 | Uncultured bacterium | 95 | EU779071.1 | **↑** |
| 12 | 66.9 | *Roseburia hominis* | 98 | NR074809.1 | **↓** |
| 42 | 13.5 | *Bacteroides thetaiotaomicron* | 99 | NR074277.1 | **↓** |
| 54 | 69.6 | *Blautia luti* | 97 | AB691576.1 | **↓** |
